# Fronto-temporal cortical grey matter thickness and surface area in the at-risk mental state and recent-onset schizophrenia: a magnetic resonance imaging study

**DOI:** 10.1186/s12888-024-05494-9

**Published:** 2024-01-09

**Authors:** Paul E. Rasser, Tim Ehlkes, Ulrich Schall

**Affiliations:** 1 Centre for Brain & Mental Health Research, The University of Newcastle, Waratah, NSW 2298 Australia; 2 Hunter Medical Research Institute, New Lambton Heights, NSW 2305 Australia; 3 Centre for Brain & Mental Health Research, McAuley Centre, Mater Hospital, Waratah, NSW 2298 Australia

**Keywords:** Magnetic resonance imaging, Clinical high risk, Psychosis, Schizophrenia, Cortex, Global functioning, Alcohol, AUDIT, Cannabis, CUDIT

## Abstract

**Background:**

Studies to date examining cortical thickness and surface area in young individuals At Risk Mental State (ARMS) of developing psychosis have revealed inconsistent findings, either reporting increased, decreased or no differences compared to mentally healthy individuals. The inconsistencies may be attributed to small sample sizes, varying age ranges, different ARMS identification criteria, lack of control for recreational substance use and antipsychotic pharmacotherapy, as well as different methods for deriving morphological brain measures.

**Methods:**

A surfaced-based approach was employed to calculate fronto-temporal cortical grey matter thickness and surface area derived from magnetic resonance imaging (MRI) data collected from 44 young antipsychotic-naïve ARMS individuals, 19 young people with recent onset schizophrenia, and 36 age-matched healthy volunteers. We conducted group comparisons of the morphological measures and explored their association with symptom severity, global and socio-occupational function levels, and the degree of alcohol and cannabis use in the ARMS group.

**Results:**

Grey matter thickness and surface areas in ARMS individuals did not significantly differ from their age-matched healthy counterparts. However, reduced left-frontal grey matter thickness was correlated with greater symptom severity and lower function levels; the latter being also correlated with smaller left-frontal surface areas. ARMS individuals with more severe symptoms showed greater similarities to the recent onset schizophrenia group. The morphological measures in ARMS did not correlate with the lifetime level of alcohol or cannabis use.

**Conclusions:**

Our findings suggest that a decline in function levels and worsening mental state are associated with morphological changes in the left frontal cortex in ARMS but to a lesser extent than those seen in recent onset schizophrenia. Alcohol and cannabis use did not confound these findings. However, the cross-sectional nature of our study limits our ability to draw conclusions about the potential progressive nature of these morphological changes in ARMS.

## Introduction

The first studies investigating differences in grey matter volume using magnetic resonance imaging (MRI) in individuals at clinical high risk of developing psychosis, known as At-risk Mental State (ARMS), date back approximately two decades ago [1, 2]. However, subsequent studies of MRI derived cortical grey matter thickness and surface area in ARMS individuals compared to healthy controls have been limited in number and have produced mixed findings [3]. A review by Luna and colleagues [4] reported inconsistent findings derived from whole brain voxel-based morphometry and functional MRI in ARMS. These inconsistencies may be attributed to small sample sizes, and to variations in several factors such as the age ranges studied, the ARMS identification criteria, the proportion of ARMS participants on antipsychotic medication when entering the study and the methods used for calculating cortical thickness and surface area from MRI data [5–17].

Some studies have reported thinner cortical grey matter in ARMS compared to healthy individuals [5–11]. However, there is minimal overlap of the affected brain regions across these studies. Dukart et al. [12] reported both increases and decreases in cortical grey matter thickness for the ARMS cohort compared to healthy participants, while several studies found no differences in cortical grey matter thickness for ARMS individuals compared to healthy controls [13–17]. One study reported smaller surface area in regions including the dorsolateral prefrontal cortex (DLPFC), medial prefrontal, para-hippocampal, fusiform, and temporal regions, in ARMS individuals compared to healthy participants [6]. On the other hand, other studies did not find any differences in surface area in ARMS individuals [11, 13].

Substance use is also a potential confounding factor in ARMS studies. Klauser et al. [16] suggested that previous findings of grey matter deficits may be linked to illicit drug use in ARMS cohorts. Alcohol and cannabis use are particularly prevalent among adolescents and young adults [18, 19]. For alcohol, MRI data from the *National Consortium on Alcohol and NeuroDevelopment in Adolescence* found significantly smaller frontal, temporal and cingulate cortical thickness in participants with high levels of alcohol consumption compared to those in a group with no or moderate alcohol consumption [20].

Cannabis is a known risk factor for psychosis which can contribute to functional decline over time [21]. Nevertheless, cannabis use alone does not appear to increase the probability of ARMS individuals developing psychosis [22]. However, the starting age of cannabis use appears to be relevant. ARMS with early onset of consuming cannabis (i.e., before the age 15 years) and with frequent use thereafter had the highest risk of transitioning to psychosis, although transitioning to psychosis was not generally higher in using versus non-using ARMS [23].This is partly supported by a meta-analysis of published data from the period of 1996 to 2015 indicating that total life-time cannabis use was not associated with ARMS transitioning to psychosis whereas current cannabis use at an abusive or dependence level increased the risk of transitioning [24]. Later Farris et al. [25] systematically reviewed the prevalence of cannabis use in ARMS and transition to psychosis published up to late 2018. They found that cannabis use was most commonly reported to be associated with transitioning to psychosis, which however, was not statistically significant for the pooled relative risk.

The cerebellum contains high densities of cannabinoid receptors [26] and regional cerebellar grey matter thinning has been reported to correlate with total lifetime cannabis consumption in adolescents and young adults not presenting with a mental illness other than cannabis abuse or dependence [27]. The study also reported that same-age first-episode schizophrenia patients, who never consumed cannabis, showed widespread grey matter reductions in the cerebellum. Also, cannabis use per se does not appear to be associated with cortical grey matter thinning in people with schizophrenia and bipolar affective disorder whereas cannabis use before the onset of mental illness is associated with reduced caudal middle frontal gyrus after controlling for tobacco and alcohol use disorders [28]. Buchy et al. [29] reported smaller hippocampal volumes in genetically high-risk individuals consuming cannabis compared to high-risk non-users. This difference became non-significant when controlling for alcohol and tobacco use. These finding emphasise the importance of controlling for the use of substances other than cannabis.

In previous studies, we found more profound cortical thinning in patients with schizophrenia (SCZ) that received antipsychotic pharmacotherapy compared to those who did not [30]. Hence, distinguishing genuine illness effects on brain structure from potential antipsychotic medication effects becomes challenging in ARMS individuals who have been prescribed with antipsychotics prior to entering a study. While there is some evidence suggesting that antipsychotics contribute to additional progressive volumetric grey matter reductions in recent onset schizophrenia patients when followed up over several years [31], the underlying driver of this observation remains unclear. Secondary metabolic effects of antipsychotics, such as weight gain, type-2 diabetes, and high blood pressure have also been associated with grey matter reductions even in the absence of symptomatic cardio-vascular disease or events (reviewed by [32]). Interestingly, in the context of ARMS, Chung et al. [6] did not find significant correlations between global measures of grey matter thickness and surface area with antipsychotic medication at the time of study. This may be expected across ARMS studies, as lower doses of antipsychotic medication are often prescribed over shorter periods of time. However, without antipsychotic treatment, some of the prodromal symptoms may be more severe and potentially surpass the psychosis threshold for ARMS. Consequently, studies that include individuals on antipsychotic medication may have included individuals at a more advanced state of illness, potentially beyond the ARMS threshold.

Reports on the associations of clinical symptoms and functional impairment with grey matter thickness and surface area in ARMS are also inconsistent. For instance, Dukart et al. [12] reported a positive correlation between cortical thickness and *Brief Psychotic Rating Scale* (BPRS [33]) in an occipital cluster for ARMS, with this region also showing significantly greater cortical thickness in controls compared to ARMS individuals.

Similarly, positive symptoms assessed using the Korean version of the *Scale of Psychosis-risk Symptoms* (SOPS [34]) subscale scores were correlated with regional thinner cortical grey matter in ARMS [9]. Furthermore, a childhood cohort study, consisting of both ARMS and healthy participants aged 17 years and younger, showed that poor premorbid function levels were associated with smaller surface area in frontal, cingulate, parietal and temporal regions [6]. On the contrary, other studies found no associations between cortical thickness and clinical and functional measures in ARMS groups [5, 8, 11, 13].

Several studies investigating cortical thickness in ARMS individuals have also included a cohort of individuals with schizophrenia [5, 8, 12, 15]. Benetti et al. [5] and Jung et al. [8] reported that the regions showing reductions in the ARMS group compared to healthy participants were similar to those observed in schizophrenia but to a greater extent. Studied focusing on schizophrenia reported that the frontal and temporal lobes are the most affected regions, characterised by thinner cortical thickness and smaller surface area compared to a healthy cohort [30].

Recently, the ENIGMA Clinical High Risk for Psychosis Working Group [35] reported widespread cortical thinning in ARMS individuals in their large-scale mega analysis of pooled MRI data. However, they did not find reduced surface areas or subcortical volumes in ARMS individuals. Moreover, they found that reduced cortical thickness in the fusiform, superior temporal and paracentral regions was associated with the conversion to psychosis whereas Del Re et al. [36] reported reduced grey matter thickness particularly in auditory and language processing regions in individuals who converted to psychosis.

In the current study we compared the cortical grey matter thickness and surface area between young ARMS individuals, recent onset schizophrenia patients, and healthy individuals. Based on the existing literature, we hypothesised (i) that the frontal and temporal lobes will show reduced regional grey matter thickness, but not surface area reductions in ARMS compared to healthy individuals and (ii) that these reductions would also be present in corresponding brain regions of recent onset schizophrenia patients, but to a greater extent than in ARMS individuals. We further hypothesised (iii) that the reductions in grey matter thickness would be more pronounced in individuals with ARMS who exhibit higher levels of symptomatology. Finally, we also predicted (iv) that symptom and function ratings, as well as the level of alcohol consumption, would be correlated with reduced frontal and temporal grey matter thickness in ARMS individuals, while levels of cannabis use would not show such correlations.

## Materials and methods

### Participants

The ARMS data for the present study were collected as a part of the *Minds in Transition* (MinT) project [37], which is a longitudinal study focused on the transition from ARMS to schizophrenia. The research was conducted in collaboration with early psychosis services located in metropolitan, regional, and rural centres across New South Wales, Australia. Participant referrals were obtained from a variety of sources, including the national *Headspace* initiative (https://headspace.org.au), mental health workers, general practitioners, school counsellors, and self-referrals.

The original MinT study recruited 102 ARMS individuals and 61 healthy control (HC) participants. In the present study, we analysed structural brain imaging data available from a subset of the MinT study, specifically 44 ARMS individuals aged 16 years and older (mean age 19.7, SD 2.1, range 16.2 – 24.1 years; 21 males and 23 females). Additionally, we included data from 19 recent onset schizophrenia patients (SCZ) who met the DSM-IV diagnostic criteria and were younger than 25 years of age (mean age 22.6, SD 1.5, range 19.9 – 24.8 years; 12 males and 7 females; mean age ARMS < SCZ: *p* < .001), which was obtained from the *Australian Schizophrenia Research Bank* (ASRB [38]). Lastly, we also analysed data from 36 HC participants (mean age 21.1, SD 2.0, range 16.6 – 24.8 years; 16 males and 20 females) pooled from both the MinT study (*n* = 17) and the ASRB (*n* = 19). The selection of participants from the ASRB was based on their age being less than 25 years.

ARMS was assessed with the *Comprehensive Assessment of At-risk Mental State* (CAARMS; version Yung et al. [39]). CAARMS defines ARMS as a significant decline of functioning over a one-year period, indicated by a drop of at least 30 points on the *Global Assessment of Function* (GAF) rating scale [40]. This decline of functioning is accompanied by (i) emerging, attenuated psychotic symptoms and/or, (ii) brief limited intermittent psychotic symptoms and/or (iii) an immediate family history of schizophrenia. In this study, the ARMS group was further divided into two subgroups based on the median split derived from a CAARMS composite score. The composite score was developed through expert consultation aiming to capture at-risk mental state as broadly as possible, thus avoiding over-reliance on individual symptom domains. Therefore, the composite score provides a better representation of the psychopathology observed in ARMS. The composite score was calculated by summing the intensity rating scores for unusual thought content, non-bizarre ideas, perceptual abnormalities, disorganized speech, alogia, avolition/apathy, anhedonia, social isolation, impaired role function, disorganising/odd/stigmatising behaviour, aggression/dangerous behaviour, mania, depression, mood swings/liability, and anxiety. The two subgroups consisted of 22 subjects with low (score ≤ 33) and 22 subjects with high at-risk symptom ratings (score ≥ 34; Table 1). The *Social and Occupational Functioning Assessment Scale* (SOFAS [40]) was also employed in the assessment.

**Table 1:** Demographic information for the healthy control and At-risk mental state (ARMS) participants as well as sub-groups. ARMS participants were divided into two groups of low and high symptomatic ARMS based on a median split of a *Comprehensive Assessment of At-risk Mental State* (CAARMS) composite score^a^. The number of participants (n) and gender male (M) and female (F), mean (m) age and standard deviation (SD) and age range in years is listed. Mean and standard deviations for CAARMS composite score, *Global Assessment of Function* (GAF), *The Social and Occupational Functioning Assessment Scale* (SOFAS), *Alcohol Use Disorders Identification Test* (AUDIT) and *Cannabis Use Disorders Identification Test* (CUDIT) is also listed

	Healthy controls	At-risk mental state	Healthy controls matched to ARMS (HC)	Low at-risk symptom ratings ARMS (LR)	High at-risk symptom ratings ARMS (HR)	Statistics
Age, n, m, SD, range years	36, 21.1 SD 2.0, 6.6 – 24.8	44, 19.7 SD 2.1, 16.2 – 24.1	29, 20.4 SD 1.5, 16.6 – 22.8	22, 19.9 SD 2.0, 16.2 – 24.0	22, 19.5 SD 2.1, 16.6 – 24.1	HC vs LR: n.s^b^. HC vs HR: n.s. LR vs HR: n.s.
Gender (M/F)	16/20	21/23	13/16	10/12	11/11	HC vs LR: n.s.^c^ HC vs HR: n.s. LR vs HR: n.s.
CAARMS composite score^a^ m, SD	-	32.3 SD 13.9	-	20.7 SD 9.1	43.8 SD 5.9	LR vs HR: *p* < 0001^d^
GAF m, SD	-	56.6 SD 11.2	-	57.6 SD 12.9	55.7 SD 9.4	LR vs HR: n.s.^d^
SOFAS m, SD	-	62.0 SD 12.1	-	64.9 SD 11.8	59.1 SD 12.0	LR vs HR: n.s.^d^
AUDIT m, SD	-	10.6 SD 9.0	-	12.2 SD 8.6	9.0 SD 9.2	LR vs HR: n.s^d^
CUDIT m, SD	-	12.8 SD 19.2	-	17.6 SD 24.1	7.9 SD 11.3	LR vs HR: n.s.^d^

*m* mean, *SD* standard deviation, *M* male, *F* female

^a^The CAARMS composite score comprised the summation of the intensity scores: unusual thought content, non-bizarre ideas, perceptual abnormalities, disorganized speech, alogia, avolition/apathy, anhedonia, social isolation, impaired role function, disorganizing/odd/stigmatizing behaviour, aggression/dangerous behaviour, mania, depression, mood swings/liability and anxiety

Statistics: ^b^*t-*Test, ^c^*X*^2^ Test, ^d^Mann Whitney U Test, *n.s.* not significant

For gender and age matching, we selected the best matched HC participants for comparison with the two respective clinical groups. We included 29 HC individuals (mean age 20.4, SD 1.5, range 16.6 – 22.8 years; 13 males and 16 females) for comparison with the ARMS groups and 26 HC participants (mean age 22.0, SD 1.5, range 19.7 – 24.8 years; 12 males and 14 females) for comparison with the SCZ group (Tables 1 and 2).

**Table 2:** Demographic information for the respective healthy control and schizophrenia participants. The number of participants (n), number of each gender male (M) and female (F), mean (m) age and standard deviation (SD) and age range in years. Mean and standard deviations of the Global Assessment of Function (GAF) for the schizophrenia group

	Healthy control participants matched to the schizophrenia group	Schizophrenia	Statistics
Age, n, m, SD, range years	26, 22.0, SD 1.5, 19.7–24.8	19, 22.6, SD 1.5, 19.9–24.8	*t* = -1.3, *p* = .2
Gender (M/F)	12/14	12/7	${\rm X}$^2^ = 1.3, *p* = .3
GAF (m, SD)	-	54.4, SD 11.0	-

*m* mean age, *SD* standard deviation, *M* male, *F* female

Exclusion criteria for the ARMS participants included pre-existing psychosis individuals whose symptoms exceeded the CAARMS psychosis threshold and individuals receiving antipsychotic pharmacotherapy. Total lifetime substance use was assessed with the *Alcohol Use Disorders Identification Test* (AUDIT [41], the *Cannabis Use Disorders Identification Test* (CUDIT [42]), and the *Opiate Treatment Index: drug use all types* (OTI [43]). Participants diagnosed with drug dependence, as assessed by either the *Structured Clinical Interview for DSM-IV Axis I Disorders* (Clinical Version; SCID-CV) or the *Kiddie Schedule for Affective Disorders and Schizophrenia for School-aged Children, Present and Lifetime Version* (K-SADS-PL), were also excluded. Additionally, participants with a history of head injury causing loss of consciousness for more than 15 min, organic brain impairment, estimated pre-morbid IQ lower than 70, impaired hearing (> 20 dB [SPL]), history of nasal trauma, or those meeting MRI exclusion criteria were also excluded from the study.

### Study protocol

Upon study entry, all ARMS participants undertook a battery of clinical and neuropsychological tests and electroencephalographic recordings over the course of 2 to 3 days (reported in [37]) which were not available for the ASRB sample [33]. Participants were also given the opportunity to participate to undergo MRI brain scans. For the first year of the study, ARMS participants were contacted every three months to assess their clinical status. At the 12-month follow-up, potential transition to psychosis was assessed by applying a DSM-IV diagnosis, using either the SCID-CV or the K-SADS-PL.

### MRI data acquisition

All MRI data used in this study were collected with 1.5 T *Siemens Avanto* MRI scanners. The acquisition protocol was consistent across the five participating sites, including the MinT and ASRB projects, which were conducted concurrently. The T1-weighted magnetisation-prepared rapid-acquisition gradient echo sequence used by all five sites employed the following parameters: a repetition time of 1980 ms, an echo time of 4.3 ms, a voxel size of 0.9765625 x 0.9765625 × 1mm^3^, and a flip angle of 15º.

### Image processing

The software *Freesurfer 5.1* [44, 45] was used to estimate the cortical thickness, surface area of the grey/white matter interface, and intracranial volume (ICV). In order to ensure data quality, we implemented a quality control process, which involved an iterative process of visual inspection, editing and re-running of *Freesurfer 5.1* as required, following the recommended protocols (http://surfer.nmr/mgh.harvard.edu/fswiki/Edits). The rigorous quality control process allowed the achievement of accurate representations of the pial and white matter boundary.

### Statistical analyses

IBM SPSS Statistics for macOS, Version 25.0 (IBM Corp. Released 2017, Armonk, NY) was used to conduct statistical analysis, including tests to examine the effects of the MRI scanner site, ICV, age and gender on the average left and right grey matter thickness and surface area of the HC participants.

The software applications mris_preproc, mri_surf2surf and mri_glmfit (*Freesurfer 6.0*) were used to perform group analyses and correlations at the vertex level with the cortical measures (grey matter thickness and surface area, respectively). The correlation analysis of grey matter thickness and surface area included symptom (CAARMS composite scores) and functional ratings (GAF, SOFAS), as well as AUDIT and CUDIT scores at the vertex level with both cortical measures.

For all surface area analyses, the ICV [46] was included as a nuisance variable. *Freesurfer 6.0* (http://surfer.nmr.mgh.harvard.edu/) was used for these analyses because the *Freesurfer 5.1* version of mris_preproc software does not apply a *Jacobian* correction for the surface area by default when transforming to the average space (target atlas, fsaverage). When using *Freesurfer 6.0*, the total quantity of surface area for each subject is conserved across the transformation to the target atlas (fsaverge [47]).

**Fig. 1:**
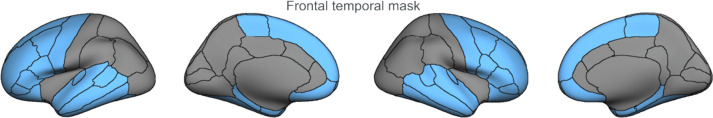
Frontal–temporal mask (shown in blue) derived from the cortical parcellation of the *Desikan-Killiany Atlas* [48]

A full-width half-maximum (FWHM) kernel of 20 mm was used for grey matter thickness and surface area analyses, together with a frontal–temporal mask to define the region of interest (Fig. 1). The frontal–temporal mask was derived from merging the frontal and temporal regions as described by the *Desikan-Killiany Atlas* [48]. The merged parcellations from the frontal lobe included the superior frontal, rostral middle frontal, caudal middle frontal, pars opercularis, pars orbitalis, pars triangularis, lateral orbitofrontal, medial orbitofrontal, precentral, paracentral, and frontal pole. The temporal lobe regions included were the superior temporal, middle temporal, inferior temporal, banks of the superior temporal sulcus, fusiform, transverse temporal, entorhinal, temporal pole, and parahippocampal.

Multiple comparison correction was performed using permutation testing with 10,000 tests, a cluster-forming threshold of .05 and a cluster-wise threshold of .05. Permutation testing was used to control for false positives that may occur with the specified parameter settings. *Monte Carlo* simulations were used to perform the permutation testing [49]. In addition, a *Bonferroni* correction was applied to take both hemispheres into account. Finally, in order to optimise the computational time and to improve the accuracy of the *p* value when clusters were found with *p* < 0.1 after 10,000 tests, permutation testing with 100,000 tests were performed.

## Results

### Immediate family history of schizophrenia and transition from ARMS to schizophrenia

Ten of the ARMS participants had a first-degree relative with schizophrenia, while 18 did not have a first-degree relative with schizophrenia and 8 participants were not aware (Table 3). CAARMS was performed at follow-up. Of the 44 ARMS participants, one transitioned to the DSM-IV diagnosis of schizophrenia. Furthermore, 14 of the ARMS participants were taking antidepressant medication upon entering the study (Table 3).

**Table 3:** Clinical information for the At-risk mental state and Schizophrenia participants

	ARMS	Low at-risk symptom ratings ARMS	High at-risk symptom ratings ARMS	Schizophrenia
1st degree relative with SCZ	10	5	5	-
Unknown details of 1st degree relative with SCZ	8	4	4	-
1st or 2nd degree relative with SCZ	-	-	-	5
Illness duration m, SD (years)	-	-	-	4.7 SD 2.3
On antipsychotics within 1 month of entry to the study	-	-	-	15
On Antidepressants upon entry / within 1 month of entry to the study	14	4	10	9
Previous treatment with Antidepressants	26	13	13	-

*m* Mean, *SD* Standard Deviation, *ARMS* At-risk mental state, *SCZ* schizophrenia

### Age and gender in ARMS and SCZ groups

There were no significant age differences between the HC (mean age 20.4, SD 1.5) and the ARMS groups (mean age 19.7, SD 2.1; *t*(71) = -1.5, *p* = 0.1). Similarly, no significant age differences were found between the less symptomatic (M = 19.9, SD = 2.0), *t*(49) = -1.0, *p* = 0.3) and the more symptomatic ARMS subgroups (M = 19.5, SD = 2.1), *t*(49) = -1.7, *p* = 0.1). Additionally, there were no significant age differences between the low and high at-risk symptom ratings subgroups for age *t*(42) = 0.6, *p* = 0.6. Moreover, there was also no significant difference in age between the respective HC group (M = 22.0, SD = 1.5) and SCZ (M = 22.6, SD = 1.5; *t*(43) = -1.3, *p* = 0.2).

The ARMS individuals were younger than SCZ subjects (*t*(61) = -5.4, *p* < .001). No significant difference in gender was observed between the ARMS and low and high at-risk symptom ratings subgroups and the HC (*X*^2^[*N* = 73, df = 1] = .06, *p* = .8, *X*^2^ [*N* = 51, df = 1] = .002, *p* < 1 and *X*^2^[*N* = 51, df = 1] = .1, *p* = .7, respectively). Similarly, there were no significant differences between the low and high at-risk symptom ratings subgroups and gender (*X*^2^(*N* = 44, df = 1] = .09, *p* = .8), and no significant differences in gender between SCZ and their respective HC group (*X*^2^[*N* = 45, df = 1] = 1.3, *p* = .3). Furthermore, higher CAARMS sub-scores were confirmed for the more symptomatic ARMS group compared to the low symptomatic ARMS group after *Bonferroni* correction for multiple comparisons (Table 4). Hence, morphological group comparisons were only calculated between groups which did not differ in age or in the number males and females.

**Table 4:** Low and high at-risk symptom at-risk mental state mean and standard deviation of CARRMS sub-scores

CAARMS item	Low at-risk symptom ARMS *n* = 22	High at-risk symptom ARMS *n* = 22	*p*^*a*^
CAARMS 1 m, SD	8.3 SD 4.1	13.0 SD 3.5	< .001
CAARMS 2 m, SD	1.8 SD 1.4	3.1 SD 1.1	.001
CAARMS 3 m, SD	1.6 SD 1.4	3.1 SD 1.1	.001
CAARMS 4 m, SD	2.8 SD 2.5	8.1 SD 3.1	< . 001
CAARMS 5 m, SD	4.5 SD 2.6	11.5 SD 3.2	< .001
CAARMS 6 m, SD	1.1 SD 2.0	4.5 SD 3.2	< .001
CAARMS 7 m, SD	7.8 SD 5.5	19.1 SS 4.1	< .001

CAARMS 1 = sum of intensity scores for Unusual Thought Content, Non-Bizarre Ideas, Perceptual Abnormalities, Disorganised Speech

CAARMS 2 = intensity scores for Attention/Concentration

CAARMS 3 = Subjective Emotional Disturbance

CAARMS 4 = sum of intensity scores for Alogia, Avolition/Apathy, Anhedonia

CAARMS 5 = sum of intensity scores for Social Isolation, Impaired Role Function, Disorganising/Odd/Stigmatising Behaviour, Aggression/Dangerous Behaviour

CAARMS 6 = sum of intensity scores for Subjective Complaints of Impaired Motor Function, Subjective Complaints of Impaired Bodily Sensation, Subjective Complaints of Impaired Autonomic Functioning

CAARMS 7 = sum of intensity scores for Mania, Depression, Suicidality & Self-harm, Mood Swings/Lability, Anxiety, OCD Symptoms, Dissociative Symptoms, Impaired Tolerance to Normal Stress

*ARMS* At-risk mental state, *m* Mean, *SD* Standard deviation

^a^*p* value from Mann–Whitney U Test, significant after *Bonferroni* correction (0.05/7 = 0.007)

### Potential scanner site effects and ICV association with cortical thickness and surface area

In the HC groups, the average cortical thickness (left H(4) = 7.4 *p* = 0.1, right H(4) = 7.2, *p* = .1) and total surface area (left H(4) = 8.7, *p* = .07, right H(4) = 8.3, *p* = .08) did not differ between scanner sites. In addition, no correlation was found between ICV and the left (*r*_*s*_ = .09, *p* = .6) and right (*r*_*s*_ = 0.1, *p* = .5) average cortical thickness in HC. By contrast, ICV significantly correlated with left and right total surface area for the HC participants (*r*_*s*_ = .89, *p* < 0.001 and *r*_*s*_ = .90, *p* < .001, respectively).

**Fig. 2:**
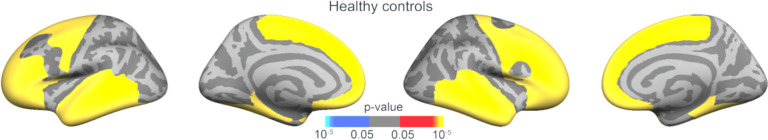
Corrected correlation maps between surface area and intracranial volume (ICV) for healthy participants using a frontal–temporal mask. Multiple comparison correction was performed using permutation testing of 100,000 tests with a cluster-forming threshold of *p* < .05 and cluster-wise threshold of *p* < .05 using a frontal–temporal mask with additional *Bonferroni* correction to take both hemispheres into account. (Orange colour = positive correlation between surface area and ICV)

At the vertex level analysis, no significant correlations were observed bilaterally between cortical thickness and ICV in HC. However, a significant correlation was confirmed bilaterally (cluster-wise *p* = .00002 for both the frontal and temporal clusters and for both hemispheres) between surface area and ICV (Fig. 2). Hence, ICV was included as a covariate in all vertex-wise analyses involving surface area.

At the vertex level, no significant correlation was found for age and no significant differences in grey matter thickness were found between males and females in the HC group. Similarly, no significant correlation at the vertex level for age or group differences between HC males and HC females were found with surface area when ICV was included as a covariate. As a result, age and gender were not included as variables for cortical thickness and surface area analyses.

### Cortical grey matter thickness and surface area in ARMS versus HC

**Fig. 3:**
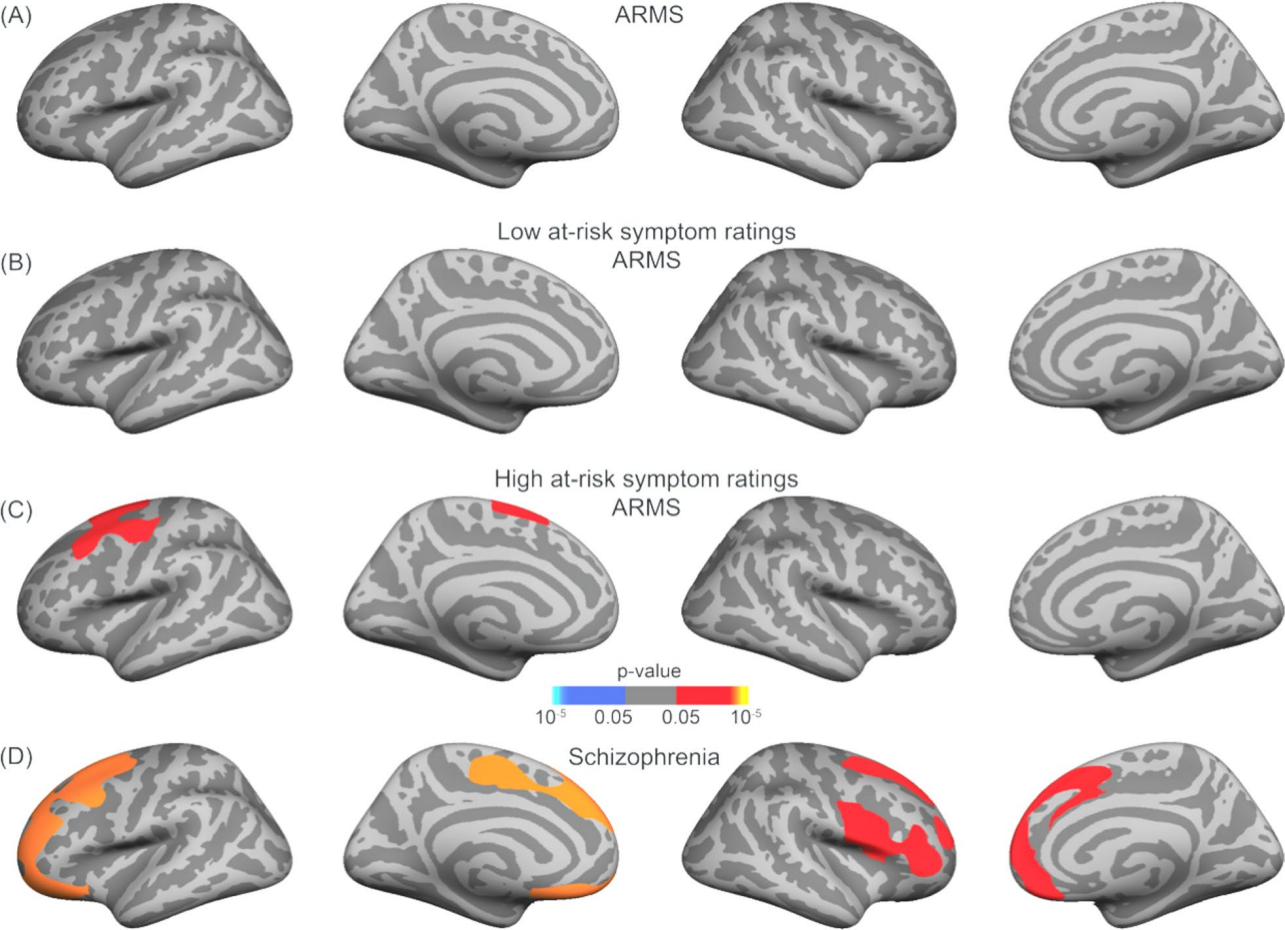
Significant grey matter thickness differences between (**A**) ARMS, **B** low symtpmatic ARMS, and **C** high symptomatic ARMS and **D** the SCZ group versus their matched HC counter parts, respectively, using a frontal–temporal mask. Multiple comparison correction was performed using permutation testing of 100,000 tests with a cluster-forming threshold of *p* < .05 and cluster-wise threshold of *p* < .05 with an additional *Bonferroni* correction to take both hemispheres into account. (Red = greater grey matter thickness in HC)

The total ARMS group and the low at-risk symptomatic ARMS subgroup did not show significant differences in cortical grey matter thickness compared to the HC group (Fig. 3A and B, respectively). In the more symptomatic ARMS subgroup, significantly reduced grey matter thickness was confirmed (*p* < .05) in a cluster found in the left hemisphere, encompassing the precentral, superior frontal, caudal, and rostral middle frontal cortex (Fig. 3C). In the right hemisphere, a non-significant cluster was found in the superior frontal region (*p* = .077).

**Fig. 4:**
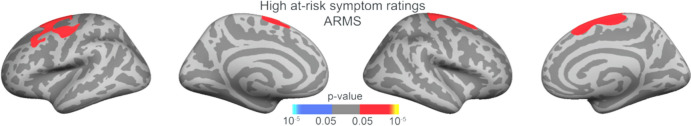
Significant grey matter thickness differences between highly symptomatic ARMS and their HC counterpart using a frontal–temporal mask. As part of the post-hoc analysis a FWHM of 15 mm was used in this case rather than 20 mm. Multiple comparison correction was performed using permutation testing of 100,000 tests with a cluster-forming threshold of *p* < .05 and cluster-wise threshold of *p* < .05 with an additional *Bonferroni* correction to take both hemispheres into account. The clusters in the left and right superior frontal regions have a *p* = .024 and *p* = .033 respectively. (Red = greater grey matter thickness in HC compared to the high symptomatic ARMS)

In the post-hoc analysis we used a FWHM of 15, instead of 20 in order to capture clusters of smaller size and greater significance. The left hemispheric cluster (*p* = .025) extended over the precentral, superior frontal, caudal, and rostral middle frontal cortex, while the right cluster (*p* = .032) included regions such as the precentral, paracentral, and superior frontal cortex (Fig. 4).

Surface area did not differ between low and high at-risk ARMS subgroups and HC.

### Cortical grey matter thickness and surface area in SCZ versus HC

The SCZ group showed a more pronounced thinning of the cortical grey matter compared to their respective HC group (Fig. 3D). A single left-hemispheric cluster was statistically confirmed (*p* < .001) for the caudal and rostral middle frontal, superior frontal, pars orbitalis, lateral orbital frontal, and precentral cortex. This cluster extended medially into the superior frontal, paracentral regions, and middle orbital frontal cortex. In the right hemisphere, two clusters of reduced grey matter thickness were observed in the SCZ group. The larger cluster covered the precentral, superior frontal, rostral middle frontal and medial orbital frontal cortex (*p* = .019) while the second cluster involved the precentral, pars opercularis, pars triangularis, pars orbitalis and rostral middle frontal cortex (*p* = .042).

Surface area did not differ between SCZ and HC control groups, thus mirroring the ARMS results.

### Cortical grey matter thickness and surface area correlations with symptom severity, function levels, and substance use in ARMS

**Fig. 5:**
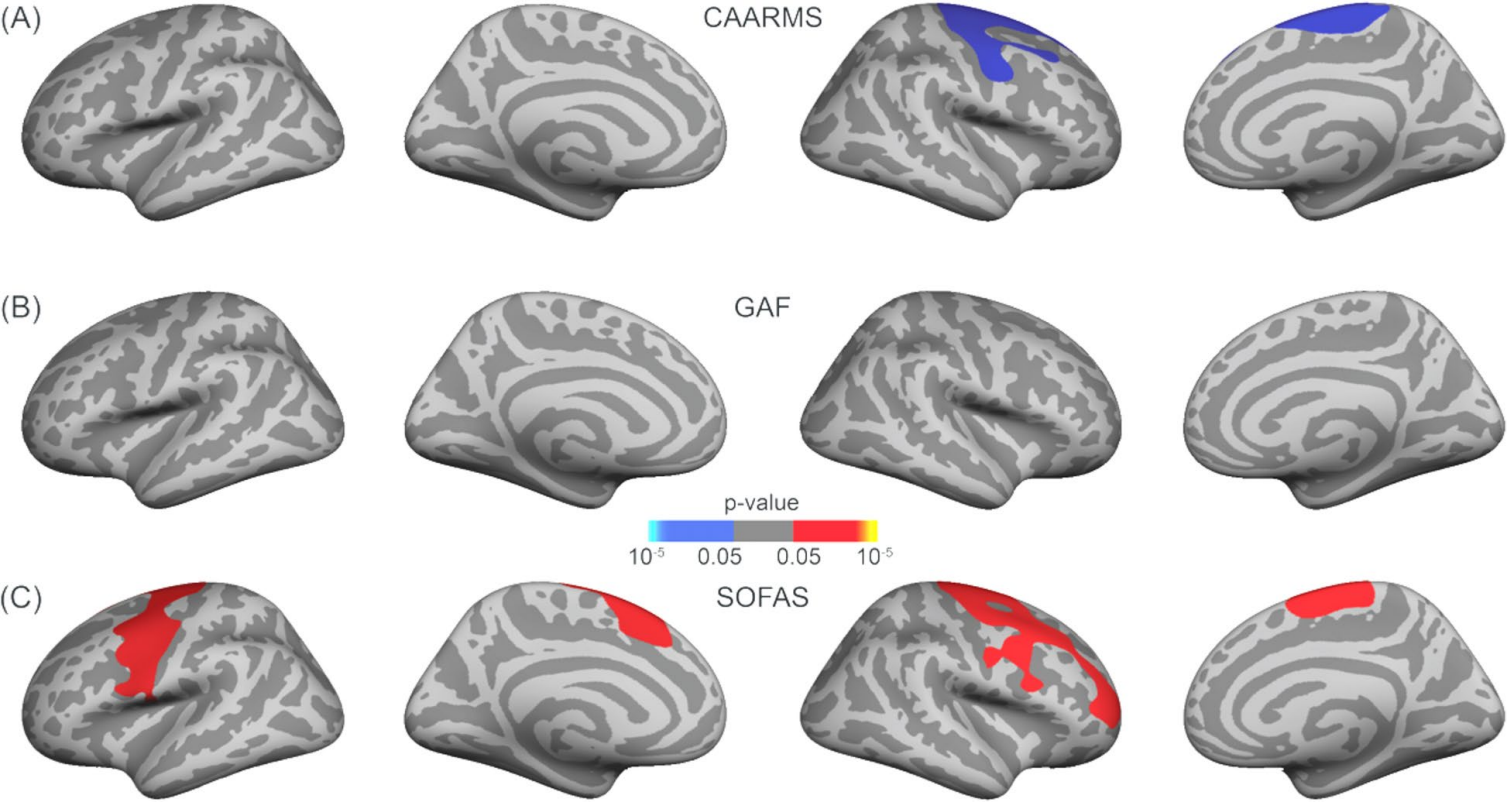
Corrected correlation maps between grey matter thickness and (**A**) CAARMS composite score, **B** GAF and **C** SOFAS scores for the ARMS cohort. A frontal–temporal mask was used, and multiple comparison correction was performed using permutation testing of 100,000 tests with a cluster-forming threshold of *p* < .05 and cluster-wise threshold of *p* < .05 with an additional *Bonferroni* correction to take both hemispheres into account. (Red = positive and blue = negative correlations between grey matter thickness and CAARMS composite and SOFAS scores)

The CAARMS composite score did not show significant correlations with GAF (*r*_*s*_ = -.23,* p* = .14) and SOFAS (*r*_*s*_ = -.21,* p* = .17) rating scores. However, there was a significant positive correlation between GAF and SOFAS rating scores (*r*_*s*_ = .77, *p* < .001). Regarding the cortical grey matter thickness in ARMS participants, significant correlations were observed with the CAARMS composite score. In the right hemisphere, a significant negative correlation (*p* = .033) was found between the cortical grey matter thickness and the CAARMS composite score, in a cluster encompassing the superior frontal, precentral, paracentral and caudal middle frontal cortex (Fig. 5A). This correlation was not confirmed for GAF ratings (Fig. 5B). On the other hand, SOFAS rating scores significantly positively correlated with cortical grey matter thickness in a single large cluster in the left hemisphere (*p* = .032), comprising the precentral, superior frontal, caudal middle frontal, and pars opercularis cortex. A similar correlation was found in a right hemisphere cluster (*p* = .017), which included the precentral, superior frontal, paracentral, caudal middle frontal, rostral middle frontal, and pars opercularis cortex (Fig. 5C).

**Fig. 6:**
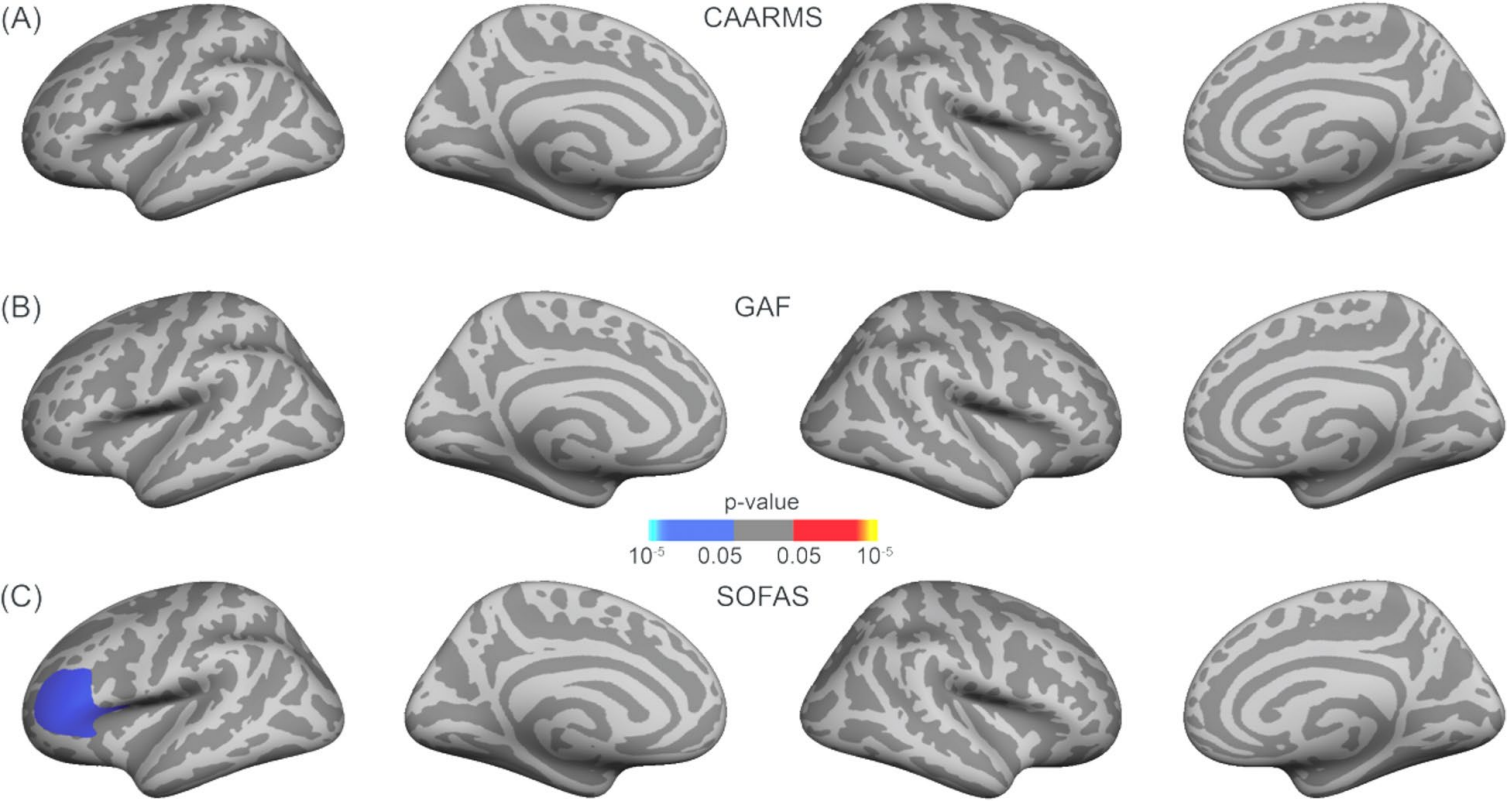
Corrected correlation maps between surface area with nuisance variable intracranial volume and (**A**) CAARMS, **B** GAF and **C** SOFAS scores for the ARMS cohort. A frontal–temporal mask was used, and multiple comparison correction was performed using permutation testing of 100,000 tests with a cluster-forming threshold of *p* < .05 and cluster-wise threshold of *p* < .05 with an additional *Bonferroni* correction to take both hemispheres into account. (Blue = negative correlation between surface area and SOFAS scores)

Regarding surface area in ARMS individuals, a significant negative correlation (*p* = .017) was found with the SOFAS score in a cluster covering portions of the left rostral middle frontal, pars opercularis, pas triangularis and lateral orbital frontal cortex (Fig. 6C). Nevertheless, no significant correlations were found between the CAARMS composite score and the GAF functional ratings with surface area (Fig. 6A and B, respectively).

The level of alcohol (AUDIT) and cannabis (CUDIT) life time use did not significantly differ between both low and high at-risk symptom ratings ARMS subgroups (alcohol use: U = 181.5, Z = -1.4, *p* = .15; cannabis use: U = 199.0, Z = -1.1, *p* = .29; Table 1). Furthermore, for the ARMS groups (including both subgroups) there were no significant correlations between the cortical grey thickness or surface area and the levels of alcohol or cannabis use. Similarly, no significant correlations were found between symptom ratings (CAARMS composite score) or function scores (GAF and SOFAS) and levels of alcohol and/or cannabis use.

### Cortical grey matter thickness and surface area correlations with function levels in SCZ

GAF ratings were the only function measure available for the SCZ sample. In this group, grey matter thickness did not correlate with GAF ratings as well as grey matter surface area when ICV was included as a nuisance factor.

## Discussion

Our study did not detect differences in fronto-temporal grey matter thickness between the at-risk mental state cohort as a whole when compared to their age-matched healthy counterparts. However, we confirmed reduced frontal and temporal grey matter thickness in the more symptomatic ARMS subgroup, as identified by median split of the CAARMS composite score.

Across the ARMS cohort, grey matter thickness was negatively associated with the level of symptom severity (CAARMS composite score), and positively with the level of socio-occupational functioning (SOFAS). We did not find any group differences in surface area between ARMS and HC groups when correcting for ICV. These findings are derived from an ARMS cohort that was not treated with antipsychotics, thereby ruling out potential confounds due to antipsychotic pharmacotherapy.

On the other hand, antidepressant pharmacotherapy was not an exclusion criterion in our study, 14 of the ARMS participants were treated with antidepressants upon entering the study. Although this represents a sizable portion of our ARMS participants, no effects of antidepressant medication dosage on grey matter thickness have previously been reported. Moreover, the potential effects of antidepressant treatment on surface area in the early stages of psychosis appears to be restricted to the inferior temporal gyrus [49].

Symptom severity and/or immediate family history of schizophrenia are key CAARMS criteria, associated with a substantial functional decline (i.e., > 30 points on the GAF rating scale over 12 months). Notably, the symptom severity CAARMS criteria, but not GAF alone, were associated with grey matter thickness in regions similar to where thinner cortical grey matter was observed for both ARMS and SCZ groups compared to HC.

CAARMS composite and SOFAS rating scores did not correlate in our study, indicating that both measures are largely independent predictors of decreased grey matter thickness in the frontal lobe in more symptomatic ARMS individuals compared to HC. In addition, the level of alcohol (AUDIT) and cannabis (CUDIT) life time consumption did not correlate with cortical thickness or surface area in ARMS. The level of alcohol and cannabis use were also independent of symptom severity in ARMS. Given that substance dependence was an exclusion criterion for this study, the potential impact of heavier substance use over longer periods of time is not covered by our sampling. However, there is also not much evidence that cannabis use in ARMS is associated with transitioning to psychosis [22].

Our findings further suggest that socio-occupational functioning (SOFA) is likely a better instrument than global functioning (GAF) when aiming to identify morphological correlates of a potential brain pathology, as it also correlates with regional cortical surface area in ARMS. Furthermore, GAF may not be an ideal measure in the context of clinical high-risk assessment, as it covers more general psychopathology. For instance, an individual with suicidality may automatically receive a low GAF score (< 20), while this same individual might function well in their daily life. In addition, we did not find a correlation between GAF score and grey matter thickness or surface area when ICV was included as a nuisance variable for the SCZ group.

Unlike some previous studies using *Freesurfer* [5, 6, 10, 13, 16, 17], we have used permutation rather than *Monte Carlo* simulation for multiple comparison correction. For some parameter settings, type 1 errors may occur using *Monte Carlo* simulation [47], and this could have contributed to the variability observed across ARMS studies. Heterogeneity across antipsychotic medications, age ranges and assessments tools may also contribute to inconsistencies.

A limitation of our study is that the MRI data was collected from five different locations. However, the scanners used (1.5 Tesla Siemens Avanto) and the acquisition protocols were identical across all locations. Potential scanner site effects were determined by phantom scans and scans conducted from the same healthy individual at each collection site. No differences were detected between scanner sites as well as when comparing the morphological measures between collections sites in the healthy control group. Unfortunately, due to the small number of participants at each collection site, any potential differences between scanners (i.e., interaction site by group) would be biased by individuals’ variations at each location. Therefore, it is impossible to separate the effects of the variation between individuals from potential scanner site effects in our analysis. This remains a study limitation.

Moreover, a direct comparison between the ARMS and SCZ groups in our study is limited by the mean age difference of 2.9 years. This is not surprising since the onset of the first episode of schizophrenia is likely to take place at a slightly older age on average than the identification of ARMS, although the onset of psychosis at a younger age is prognostically worse [50].

Another limitation is the particularly low one-year transition rate from ARMS to SCZ in our study, which was only 2.3% due to only one individual developing schizophrenia. This individual was also the only study participant from the ARMS cohort who commenced antipsychotic medication while in the study. The transition rate may be underestimated due to the loss of contact with three other study participants during the follow-up period. Notwithstanding, the transition rate in our study is considerably low when compared to other reports (reviewed by [51]), although it was higher in the original MinT study with 7 out of 67 participants, or 10.4% [37]. This is likely a result of our strict exclusion criteria, such as not including ARMS individuals who were being treated with antipsychotics. Hence, our study is most likely to be representative of less symptomatic ARMS individuals compared to other studies that include individuals already receiving antipsychotic pharmacotherapy.

In the past decade, a noticeable decline in transition rates from ARMS to first-episode psychosis has been observed [52]. Improvements in psycho-education, better referral pathways, and early intervention have been discussed as potential factors contributing to progressively declining transition rates [53]. The original MinT study reported a significant improvement of general psychopathology in the ARMS cohort as the study progressed [38]. The specific reasons for this improvement remain unclear. However, for the use of antidepressant pharmacotherapy and the participation in a study may have had a beneficial impact.

Treatment status aside, Merritt et al. [51] conducted a systematic review of longitudinal MRI data in ARMS. They found that while grey matter thinning is part of the normal brain maturation process in healthy individuals, particularly in the crucial age range where ARMS identification and transition to psychosis generally occurs [54, 55], accelerated grey matter decline in the temporal, cingulate and parietal cortex was found across the ARMS studies reviewed. This grey matter decline was further pronounced in individuals who remained symptomatic or eventually developed psychosis. The authors concluded that longitudinal structural imaging data are more sensitive in predicting transitions from ARMS to psychosis than cross-sectional data. However, the putative pathological mechanisms driving the accelerated cortical grey matter reduction during the prodromal phase of schizophrenia are still unknown, thus being an important target for future research.

## Conclusions

Our findings support previous research reporting thinner frontal grey matter in ARMS individuals. Importantly, this association was observed in an ARMS cohort that was not treated with antipsychotic medication, suggesting that our observations were not confounded by such treatment. Notably, recreational (i.e., non-addictive) alcohol and cannabis consumption was not associated with cortical thickness or surface area measures. The regional extent of the cortical thinning in ARMS was similar to that found in young recent onset SCZ patients, but this similarity was only evident in the subgroup of ARMS individuals who were more symptomatic. Furthermore, a thinner cortex in the frontal lobe was associated with increasing symptom severity and poorer socio-occupational functioning, which provides further evidence of the association between cortical thinning and the key criteria defining ARMS. However, future research is needed to deepen our understanding and the clinical implications of cortical thinning in ARMS.

## Data Availability

MinT study data are archived at the University of Newcastle and available from the corresponding author on reasonable request with institutional ethics approval. ASRB data are available from Neuroscience Research Australia (NeuRA; https://neura.edu.au).

## Abbreviations

AUDIT: Alcohol Use Disorders Identification Test
ARMS: At Risk Mental State
ASRB: Australian Schizophrenia Research Bank
BPRS: Brief Psychotic Rating Scale
CUDIT: Cannabis Use Disorders Identification Test
CAARMS: Comprehensive Assessment of At-risk Mental State
FWHM: Full-Width Half-Maximum
GAF: Global Assessment of Function
HC: Healthy Control
ICV: Intracranial Volume
K-SADS-PL: Kiddie Schedule for Affective Disorders and Schizophrenia for School-aged Children, Present and Lifetime Version
MRI: Magnetic Resonance Imaging
MinT: Minds in Transition
SOPS: Scale of Psychosis-risk Symptoms
SCZ: Schizophrenia
SOFAS: Social and Occupational Functioning Assessment Scale
SCID-CV: Structured Clinical Interview for DSM-IV Axis I Disorders
